# Differential Antimicrobial Effect of Three-Sized Biogenic Silver Nanoparticles as Broad-Spectrum Antibacterial Agents against Plant Pathogens

**DOI:** 10.3390/antibiotics12071114

**Published:** 2023-06-28

**Authors:** Munirah F. Aldayel, Nermin El Semary, David G. Adams

**Affiliations:** 1 Biological Sciences Department, College of Science, King Faisal University, Al-Ahsa 31982, Saudi Arabia; nelsemary@kfu.edu.sa; 2 Botany and Microbiology Department, Faculty of Science, Helwan University, Cairo 11795, Egypt; 3 Faculty of Biological Sciences, Leeds University, Leeds LS2 9JT, UK; d.g.adams@leeds.ac.uk

**Keywords:** antimicrobial, fruits, environmental sustainability, plant pathogenic bacteria, cyanobacteria, silver nanoparticles

## Abstract

Background: Massive fruit losses are caused by microbial pathogens of unknown identities. Therefore, ecofriendly biocontrol measures are well sought after, and biogenic silver nanoparticles are plausible candidates. Here we investigate the antimicrobial effect of three different sized AgNPs samples on those pathogens. Methodology: Identities of three local pathogenic bacteria were investigated using molecular methods. Three different-sized samples of silver nanoparticles were bio-synthesized in the external solution of a cyanobacterial culture, characterized, and used in antimicrobial bioassay. Results: The pathogens were identified as *Erwinia pyrifoliae*, *Staphylococcus warneri,* and *Xanthomonas citri*. UV-vis. and FTIR spectroscopy confirmed the biosynthesis of AgNPs. and their three different sizes were confirmed using Scanning electron microscopy. Growth of bacterial pathogens was inhibited by all three samples of AgNPs, but the largest inhibition zone was for the smallest sized AgNPs against *Staphylococcus warneri* (1.7 cm). Discussion: The identity of the pathogens infecting different local fruits is reported for the first time. They belong to different bacterial lineages. The fact that biogenic AAgNPs were effective against all of them shows their broad-spectrum of antibacterial effect. Customized biosynthesis was successful in yielding different-sized AgNPs. The smaller the AgNPs, the stronger the antimicrobial impact. Conclusion: Local bacterial species infecting fruits are diverse. Customized biogenic AgNPs are effective broad-spectrum biocontrol agents against bacterial pathogens of local fruits and thereby help maintain food security and environmental sustainability.

## 1. Introduction

Nanoparticles are minute particles of size 10^−9^ m whose chemical and physical characteristics are different from bulk particles [1]. Cyanobacteria are wide-spread photosynthetic prokaryotes that represent bio-factories for the production of nanoparticles [2,3]. Cyanobacteria grow rapidly in inexpensive growth media, require only minerals and light energy for growth and can produce nanoparticles without toxic byproducts or energy consumption [4,5]. Therefore, their synthesis of nanoparticles is both cost-effective and eco-friendly [2]. Currently, there is a surge to find new biological sources for antimicrobial agents. In this regard, cyanobacteria are considered promising sources of silver nanoparticles that are effective antimicrobial agents [4,5]. They are reported to reduce silver ions via chemical entities or reductase enzymes such as NADH-dependent reductases and electron carriers such as quinones [6]. The shape and size of the nanoparticles vary according to the synthetic methods, reductant, and biological source [3,7]. Hong et al. [8] used the microwave-assisted method for the preparation of three different shapes of silver nanoparticles: nanocubes, nanospheres, and nanowires. They found that silver nanowires exhibited the weakest antibacterial activity due to poor contact with the bacterial cells as compared with silver nanocubes and nanospheres. Silver nanocubes showed stronger antibacterial activity than nanospheres. They concluded that the shape effect of AgNPs is attributed to the specific reactivity of the surface areas and facets. Indeed, [9] reported that nanoscale size and the presence of a {111} plane increase the biocidal action of silver nanoparticles. With regard to the effect of size, [10] showed that the smaller the size of nanosilver particles, the more effective they are as antibacterial agents. However, little is known about the biogenically-synthesized nanosilver particles and the relation between their size, shape, and their antimicrobial activity, as well as the extent of their antimicrobial bioactivity whether wide-spectrum or narrow. Recently, Oves et al., 2023 [11] showed that by using the flower extract of *Bougainvillea glabra* as a biological reducing agent, silver nanoparticles were generated. The biological synthesis of biogenic silver nanoparticles may proceed internally and externally and enzymes [12], such as NADH-dependent nitrate reductase, act as an electron shuttle by taking electrons from nitrate molecules and transforming them into silver metal ions leading silver nanoparticles formation. Also [13] suggested that the negatively charged carboxylic group in bacterial cell walls may provide an electrostatic interaction and reduction. Moreover, some amino acids such as arginine, aspartic acid, cystine, glutamic acid, lysine, and methionine may be involved. They act as catalysts and produce hydroxyl ions that react with the reducing agent’s aldehyde. Interestingly, [14] showed that biogenic silver nanoparticles generated through fungi-mediated synthesis were small (15.56 ±  9.22 nm), spherical, and stable (zeta potential of—38.43 mV) AgNPs with good crystallinity with the FTIR spectroscopy indicating the presence of hydroxyl, amino, and carboxyl groups, on the surface of silver nanoparticles which in turn showed antimicrobial and antibiofilm activities against Gram-positive and Gram-negative bacteria. In addition, they found the MIC and MBC values to range between 16–64 and 32–512 μg mL^−1^. Using cyanobacterial extract from novel cyanobacterium called *Desterifilum* sp., Hamida et al., 2020 [15] performed green synthesis of silver nanoparticles by adding AgNO_3_ to cyanobacterial filtrate at room temperature under direct illumination (2000 ± 200 Lux) for 24 h. Indeed, silver nanoparticles were formed which exhibited antimicrobial activity against human pathogenic bacteria. However, they used a single concentration of the precursor silver nitrate and they prepared the cyanobacterial filtrate by filtering through a Whatman filter No. 42. The shape of the biosynthesized silver nanoparticles was spherical but with some aggregation and they suggested that polysaccharides and proteins may have contributed to the bio-fabrication of the synthesis of silver nanoparticles. In the present study, we aim to investigate the effect of biosynthesized silver nanoparticles that were biosynthesized by three different concentrations of silver nitrate. Additionally, the cell-free filtrates were prepared by filtration using micromillipore filtration units. The resulting different sized silver nanoparticles were used as antimicrobial agents against different plant pathogenic bacteria. Those different plant pathogens (both gram-positive and gram-negative) were isolated and identified using molecular methods as part of the study. The factors of the size and shape of silver nanoparticles are also addressed. Antibiosis is addressed using several tests to select for the most efficient silver nanoparticle size as antimicrobial agents. Moreover, the study also documents some of the different plant pathogenic bacteria found in Al Ahsa, Kingdom of Saudi Arabia.

## 2. Materials and Methods

Materials used in this investigation including silver nitrate and other reagents mostly purchased from Sigma. The bacterial strains were isolated from different infected fruits bought from the local market in Al-Ahsa, KSA. Symptoms of infection included the following: (1) Apple: The fruit was shriveled and brown; (2) Dates: fruit had spots and (3) Mandarin had large corky lesions with centered cracks on a fruit.

### 2.1. Isolation of the Cyanobacterial Strain and Pathogenic Bacteria

Water samples were collected from rice field Al Ahsa, centrifuged at 3000 rpm to get rid of some of the contaminating bacteria, the cyanobacterial biomass was spread on solid BG 11 medium. The agar streaking was repeated several times and the isolated colonies were used to establish a liquid culture. The culture was subjected to antibiotic treatment (ampicillin 200 uL/L) in the dark overnight. The culture was washed with BPS, recentrifuged and the biomass used to establish a liquid culture. The last two steps were repeated until an axenic culture was obtained and identified by light microscopy [16]. On the other hand, the process of serial dilution was used to isolate pathogenic bacteria from fruits. The fruits were smashed into a suspension with BPS in a pre-sterile mortar and pestle, and the mixture was serially diluted from 10^−1^ to 10^−6^ CFU/mL. A spreader was used to spread 100 μL of each diluted fruit suspension onto nutrient agar medium that was clearly labeled. Pure colonies of bacteria were inoculated into 70% broth media (HIMEDIA) containing 30% Glycerol (*v/v*), incubated at 30 °C for 24 h, and then kept at 4 °C. To isolate bacterial DNA, isolates were grown in 5 mL tubes containing 2 mL of Luria broth. To precipitate bacterial pellets, 2 mL of bacterial suspension was centrifuged at 5000 rpm for 5 min.

### 2.2. DNA Extraction

DNA was extracted according to [17] as follows: 20 mg of freshly harvested bacterial pellet was ground with 500 μL of Dellaporta buffer (100 mM Tris pH 8, 50 mM ethylenediaminetetraacetate EDTA, 500 mM NaCl, 10 mM beta-mercaptoethanol, BME). A total of 33 μL of 20% sodium dodecyl sulfate (SDS, *w*/*v*) was added, vortexed and incubated for 10 min at 65 °C. Moreover, 160sixty μL of 5 M potassium acetate was added, vortexed and centrifuged for 10 min at 10,000 rpm. Following which, 450 μL of supernatant was transferred to a new tube and 450 μL phenol, chloroform and isoamyl-alcohol (PCI) 25:24:1 was added. The mixture was vortexed then centrifuged for 5 min at 10,000 rpm. After, 400 μL of the upper phase was removed and added to 0.5 volume of isopropanol and centrifuged for 10 min at 14,000 rpm. The nucleic acid pellet, after discarding the supernatant, was washed with 70% ethanol and centrifuged for 5 min at 10,000 rpm. The pellet was resuspended in 100 μL ddH_2_O. 

### 2.3. Polymerase Chain Reaction (PCR)

Amplification of the 16S rRNA gene from bacterial isolates was carried out using the universal primers; 27F 5′-AGA GTT TGA TCM TGG CTC AG-3′ and 1492R 5′-TACGGYTACCTTGTTACGACTT using the following program; initial denaturation at 94 °C for 5 min, 30 cycles of the amplification using the following stages; denaturation at 94 °C for 45 s, annealing at 55 °C for 60 s, and extension at 72 °C for 60 s in 30 amplification cycles, followed by a final extension step at 72 °C for 10 min. PCR was done in a 25 µL reaction containing 1 µL of the DNA extract (40 ng of total DNA), 2 mM MgCl_2_, 2.5 µL of 10× PCR buffer,1.5 µL of 10 µM of each primer, 2.5 µL of 10 mM dNTPs, 0.3 µL of 5U Taq DNA Polymerase and the reaction was made to 25 µL with nuclease-free water. PCR products were purified using the QIAquick^®^ PCR purification kit according to the manufacturer’s instructions. The purified PCR products were sequenced by Macrogen Inc. (Seoul, Republic of Korea), and sequencing of the purified isolates was performed in both directions using a primer pair. The sequences were uploaded on BLASTn of Genbank and a similarity check was run. For analysis of phylogenetic inference, the closely related sequences were used alongside the sequences retrieved for all pathogenic bacteria to establish phylogenetic trees using the tree construction within BLASTn.

### 2.4. Biosynthesis of Silver Nanoparticles by Cyanobacterial Cultures

**Scheme 1:**
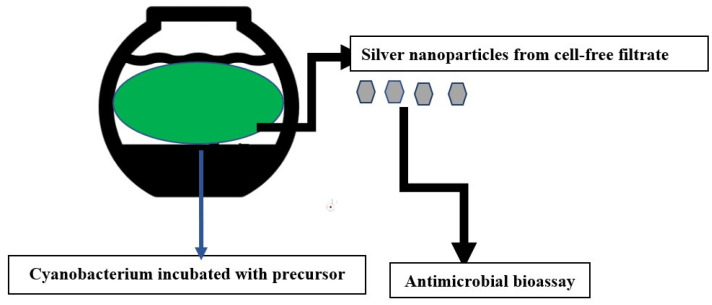
Method of preparation.

To synthesize silver nanoparticles of different sizes, the method of [17] was followed (Scheme 1). Briefly, 5 ml of mid-logarithmic *Cyanothece*-like sp. culture diluted with BPS to 18 mL was incubated with 0.2, 1, or 2 mL of 10 mM silver nitrate solution and the mixture was completed to 20 mL. The samples were allowed to stand in the dark at 25 °C until the appearance of brown colour, but with varying intensity for the three treatments. This colour is indicative of the formation of silver nanoparticles. The external solution of the cyanobacterial cultures was filtered through millipore filters of a pore diameter size of 0.2 µm.

### 2.5. Characterization of Silver Nanoparticles

The steps detailed in [17] were followed. The plasmon resonance absorption spectrum was determined using a UV-visible spectrophotometer in the wavelength range 200–600 nm. FTIR chemical functional groups were identified by the banding pattern in the FTIR spectrum. 

### 2.6. Scanning Electron Microscopy

Synthesis of different-sized silver nanoparticles was confirmed by Scanning electron microscopy. Both control and aqueous external solutions that were filtered from the three treatments of biogenic nanosilver were examined by SEM. A drop of each sample was mounted on metallic stub, air dried, and fixed using double face adhesive carbon. Sputter-coating with gold was carried out three times to increase conductivity, reduce thermal damage, and improve secondary electron signals. Samples were visualized using a Joel JSM-5510LV scanning electron microscope. 

### 2.7. Antimicrobial Bioassay

Two pathogenic bacteria were isolated from infected apple and mandarin fruits. They were purified triple times and kept as pure cultures for identification by molecular methods using PCR, sequencing, and phylogenetic inference. The third bacterium was previously characterized as the gram-positive *Staphylococcus warneri* that was isolated from date fruit. The antibacterial potency of silver nanoparticles biosynthesized by *Cyanothece*-like sp. was evaluated against pathogenic bacteria. The sensitivity of pathogenic strains to the extract was assayed through a modified Kirby Bauer Disk Diffusion Susceptibility method. Sterilized paper discs were saturated with 30 µL of a silver nanoparticle suspension. Discs were dried and placed on the surface of a nutrient agar medium which was inoculated with bacterial suspensions prepared in physiological saline. Plates were kept for 2 h at 4 °C to ensure diffusion of bioactive material, after which, the plates were incubated at 37 °C. Discs containing 30 µL of sterilized distilled water were left to dry and used as negative controls, whereas positive control sets included discs of the antibiotic chloramphenicol at a concentration of 50 µg/L. Plates were incubated for 24 h and the diameter of inhibition zones (mm) were measured in triplicate and the average standard deviation was recorded. Statistical analysis (one-way ANOVA) was performed using Minitab package, version 13, to evaluate the effect of size of biogenic silver nanoparticles on their antimicrobial activity. To ascertain the antibacterial activity of silver nanoparticles, several methods were used [18,19].

#### 2.7.1. Antibacterial Action and Measurement of Minimum Inhibitory Concentration (MIC)

In dimethylsulfoxide (DMSO, 0.1%), silver nanoparticles 9.2 × 10^−7^ M were placed. Disks made of filter paper containing silver nanoparticles each were utilized in the experiment. After diluting the tested bacterial species’ culture to 1 × 10^6^ CFU, the diameter of the inhibition zones was measured to assess the silver nanoparticles’ antibacterial activity. Using the serial dilution approach, the minimum inhibitory concentration (MIC) was determined according to [20].

#### 2.7.2. Time-Kill Test of AgNPs

For *Erwinia amylovora*, *Staphylococcus warneri*, and *Xanthomonas citri*, AgNPs were diluted with the nutritional broth medium (containing 1.5 × 10^8^ CFU/mL bacterial inoculum) to concentrations of 0 × MIC, 0.5 × MIC, 1 × MIC, 2 × MIC, and 4 × MIC. A volume of 0.1 mL of the medium was grown on Müller-Hinton agar and incubated at various times (0, 0.5, 1, 2, 4, and 6 h) for 24 h at 37 °C. All experiments were performed in triplicates. 

## 3. Results

### 3.1. Molecular Characterization and Phylogenetic Inference of Pathogenic Bacteria

**Figure 1:**
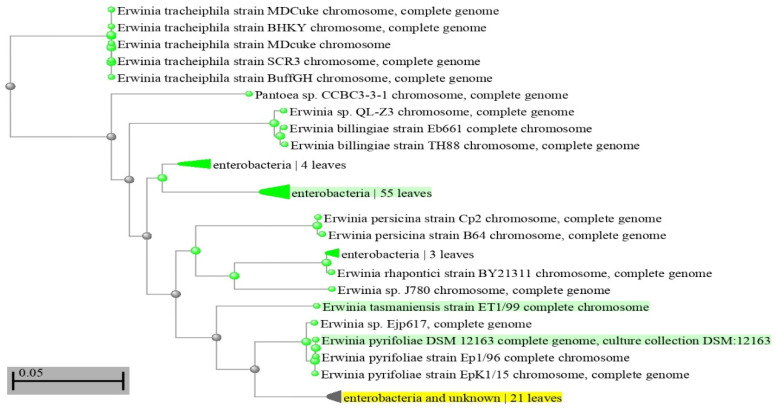
A Neighbor-joining phylogenetic tree for *Erwinia pyrifoliae (pyrifoliae).* The sequence retrieved is highlighted in yellow. Bootstrap value is indicated by the scale bar. The tree was constructed using the Phylogenetic tree reconstruction tool in BLASTn.

**Figure 2:**
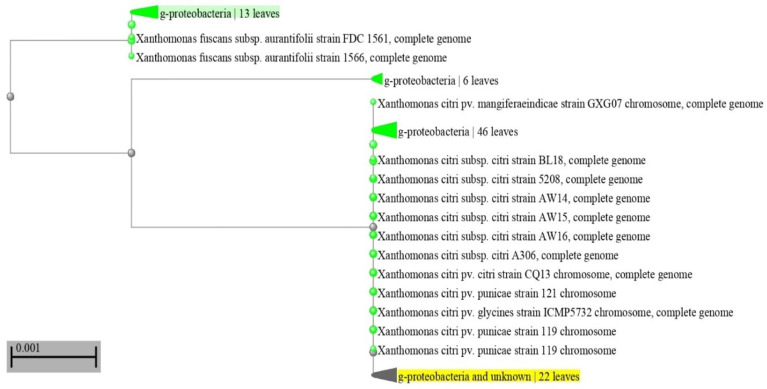
A Neighbor-joining phylogenetic tree for *Xanthomonas citri.* The sequence retrieved is highlighted in yellow. Bootstrap value is indicated by the scale bar. The tree was constructed using the Phylogenetic tree reconstruction tool in BLASTn.

The bacterium isolated from apples was identified as the gram-negative rod-shaped bacterium *Erwinia pyrifoliae* whereas that from mandarins was identified as *Xanthomonas citri,* which is a gram-negative, rod-shaped proteobacterium. The Neighbor-joining phylogenetic tree showed the co-clustering of the first isolate with strains of *Erwinia pyrifoliae* thereby confirming its identity (Figure 1). Similarly, a Neighbor-joining phylogenetic tree (Figure 2) confirmed the identity of *Xanthomonas citri*.

### 3.2. UV-Visible Spectroscopy 

**Figure 3:**
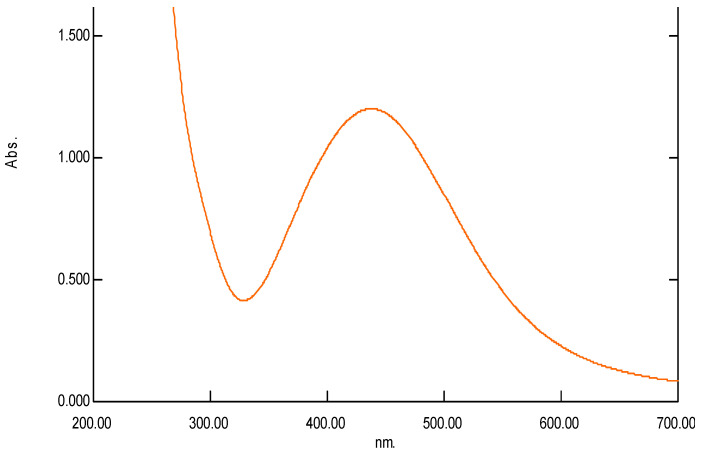
UV-visible spectrum of the largest sized biogenic silver nanoparticles (average size 92 nm). The X axis denotes wavelength, whereas the Y axis denotes absorbance.

The formation of silver nanoparticles in the cell-free filtered external solution was confirmed by the UV- visible absorbance spectra of the three samples. The three samples of biogenic nanosilver showed a strong absorbance peak at 450 nm (Figure 3 and Supplementary Materials, Figures S1 and S2). This is within the range of plasmon resonance that is characteristic of silver nanoparticles [3]. The characteristic plasmon resonance of silver nanoparticles is due to the vibrational modes of electrons based on the size and shape of nanoparticles [17]. 

### 3.3. FTIR Spectroscopy

The Fourier transmission infrared spectrum of silver nanoparticles showed the characteristic signal of silver nanoparticles present at 3356–3350 cm^−1^ overlapping with the OH signal, possibly resulting from phenolic compounds leaking from cyanobacteria. Moreover, at 1636–1637 cm^−1^ there was a clear C-H stretching (Supplementary Materials, Figures S3–S5). No other functional groups were detected. The pattern of spectrum of silver nanoparticles is similar to that reported by Zhang et al. (2016) [21], thus confirming the formation of silver nanoparticles. With regard to the confirmation of corona absence, the lack of bands at 1209 cm^−1^ and at 1533 cm^−1^ indicated its absence. The presence of signals at wave numbers of 1427, 1271, and 842 cm^−1^ corresponds to the C–C in the ring, C–N stretching and C–C in ring vibrations of covalent bonds, respectively, most likely related to the extracellular polymeric substances such as polysaccharides and peptides (Mota et al., 2021. [22]. The peak at 513 cm^−1^ indicates the vibration frequency of the Ag-O ionic bond group according to Gharibshahi et al., 2017) [23]. We indeed detected a signal within that range as in Supplementary Materials Figure S3.

### 3.4. Scanning Electron Microscopy

SEM was used to detect particle shape and size. The electro-micrograph confirmed the angular nature of the AgNPs (Supplementary Materials; Figure S6A–C). The Size detected by SEM was congruent with the size previously reported by Dynamic Light Scattering [13] when using the same synthesis method detailed in El Semary and Bakir, 2022 [17] where the average of the three size ranges was 34 nm for the smallest, 67 nm for the medium, and 93 nm for the largest nanoparticles.

### 3.5. The Antimicrobial Bioassay of Biogenic Nanosilver Samples against Plant Pathogenic Bacteria

The three samples of biogenic silver nanoparticles were analysed for their ability to adversely inhibit these diverse pathogenic bacteria in order to investigate their broad-spectrum antimicrobial activity. The antimicrobial bioassay showed broad spectrum action of silver nanoparticles of different sizes. They were effective against gram-positive *Staphylococcus warneri* and gram negative *Xanthomonas citri* and *Erwinia pyrifoliae (*Table 1). The statistical analysis showed significant differences among the different treatments as compared to negative control. All three samples of silver nanoparticles were effective as antimicrobial agents against the three tested local plant pathogens but with varying degrees of efficacy. The smallest sized silver nanoparticle was the most active. This is in total agreement with what Dong F. et al. [20] reported that AgNPs were effective at different sizes and concentrations and that the smaller the particle size, the greater the antibacterial activity.

**Figure 4:**
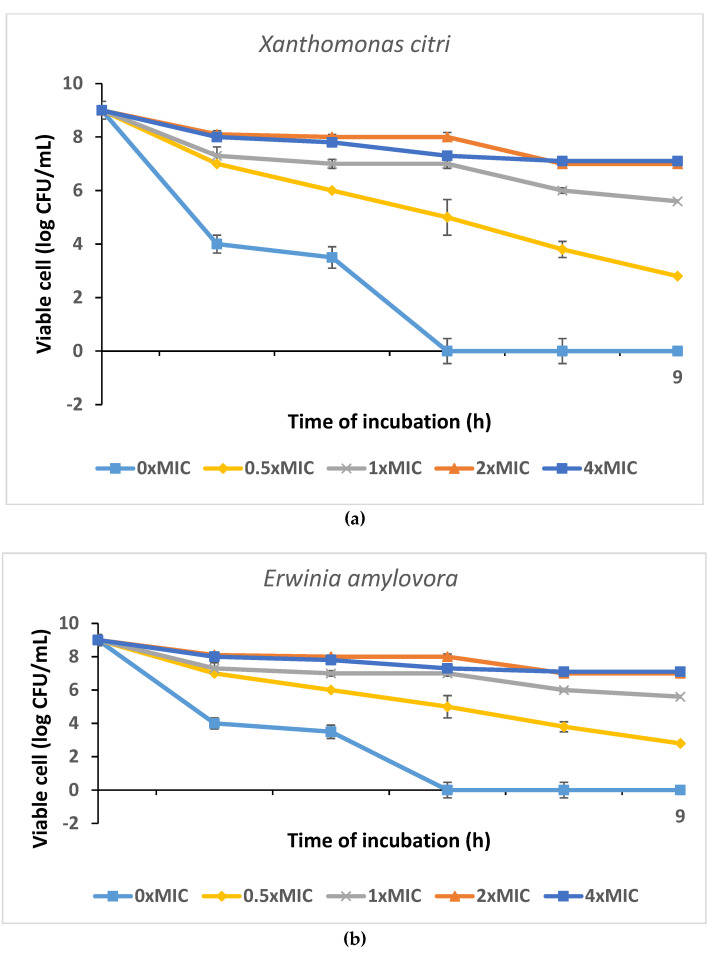

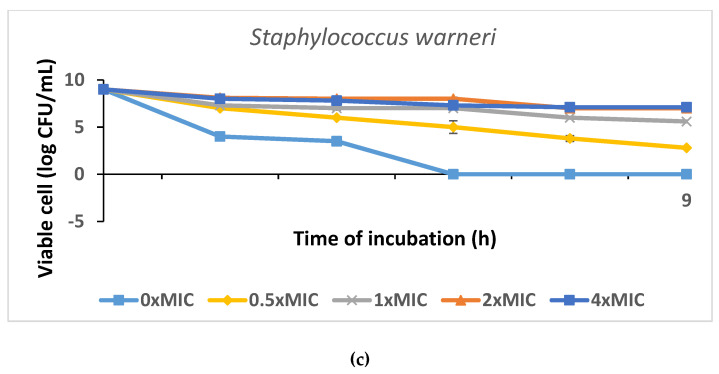
(**a**–**c**)**.** The time-kill curve plots of *S. enteritidis*, after the exposure to the PT-AgNPs at 0 × MIC, 0.5 × MIC, 1 × MIC, 2 × MIC, and 4 × MIC.

The results of the disc diffusion method-based antibacterial test of the PT-AgNPs against *Erwinia amylovora, Staphylococcus warneri, and Xanthomonas citri* are shown in Figure 4 and Supplementary Materials Figure S7. Except for *Staphylococcus warneri*, for which the MIC was 50 g/mL, the data showed that PT-AgNPs had outstanding antibacterial efficacy against all tested bacterium strains. Additionally, the outcomes of the time-kill studies demonstrated that all the examined bacteria were killed by the AgNPs at 4 MIC after 2 h (Figure 4, Table 2, Table 3 and Table 4).

Data are expressed as the mean zone of inhibition in mm followed by SD. The values with different subscript letters in the same column and those with different superscript letters in the same row are significantly different according to an ANOVA and Duncan’s multiple range tests.

## 4. Discussion

Molecular and phylogenetic analyses confirmed the identity of *Erwinia pyrifoliae,* which is a gram-negative bacterium that causes shoot blight, a disease that affects trees of apples and pears. The disease can kill fruit, shoots, and entire trees. The molecular and phylogenetic analyses also confirmed the identity of *Xanthomonas citri* which is a gram-negative bacterium that causes citrus canker. *Staphylococcus warneri* on the other hand is a gram- positive bacterium. These pathogenic bacteria, alongside the previously identified *Staphylococcus warneri,* were test organisms in the antimicrobial bioassay using biogenic silver nanoparticles. The biological synthesis of nanoparticles helps avoid the chemical and physical synthetic methods with their lengthy procedures and hazardous by-products. Biological synthesis also allows the cost-effective synthesis of nanoparticles at physiological pH which renders them biocompatible with cellular systems [17,20]. Cyanobacteria have been found to be an eco-friendly, cost-effective bio-factory of biogenic nanoparticles [4]. Indeed, the *Cyanothece*-like isolate used here in the current study proved to be an efficient nanoparticle producer and was employed to biosynthesize three different sized silver nanoparticles efficiently. The filtration of the external solution using Millipore filters of 0.2 µm pore diameter resulted in the removal of macromolecules mostly leaving ions in the cell free filtrate. According to [7], those ions are involved in the reduction of silver ions and formation of silver nanoparticles. The success in the synthesis of three-sized silver nanoparticles was confirmed by chemical analyses including UV, FTIR and DLS as well as SEM. The UV-Visible spectrum showed the characteristic plasmon resonance of silver nanoparticles is due to the vibrational modes of electrons based on the size and shape of nanoparticles [17].

The lack of functional groups associated with silver nanoparticles as FTIR results show indicates the effectiveness of filtration in preventing macromolecules from associating with silver nanoparticles, thereby preventing the formation of corona. This corona usually develops from interfacial interactions between silver nanoparticles and biological fluids including proteins. Corona formation can lead to limited cytotoxicity of silver nanoparticles where it interferes with the dissolution of AgNPs to toxic silver ions [24,25]. In our protocol, the microfiltration was apparently effective in preventing corona formation and hence the cytotoxicity of AgNPs was preserved. Nevertheless, the FTIR consistently showed the characteristic band indicative of AgNPs which overlaps with the OH band [17].

A scanning electromicroscopic analysis was used (SEM) for the determination of shape and size. SEM showed that silver nanoparticles were angular in shape. and this may contribute to the high broad spectrum bactericidal effect. Indeed, it was reported that silver nanocubes showed greater antibacterial effect against *Escherichia coli* (Gram negative) as compared to silver nanospheres [8]. Previously, DLS analysis revealed the size range for the smallest sized sample which was derived from 0.2 mL AgNO_3_ was of average 33.9 nm, the middle with an average of 67 nm, and the largest average was 92.5 nm [17]. The synthesis of three sizes of silver nanoparticles was further confirmed in this study by scanning electron microscopy which also confirmed the shape of the produced particles. The biogenic AgNPs had angular form and their size coincided with the DLS results previously reported [17].

The proposed mechanisms by which cyanobacteria produce silver nanoparticles was the focus of several studies. For example, Ali et al. [26] reported that filamentous cyanobacteria reduce silver ions using ß-NADPH-dependent nitrate reductase. Lengke et al. [4] reported that the biosynthesis of silver nanoparticles in cyanobacteria occurs both inside the cells (with size < 10 nm) and in solution (with size 1–200 nm), forming spherical and octahedral platelets over the time course. This is in agreement with our results where the different sized nanoparticles in the external solution fell within the range reported by [4]. The shape of our particles also corresponds to the octahedral platelets [4].

Ferdous and Nemmar [24] reviewed the proposed mechanisms of antibacterial action of the silver nanoparticles. One scenario proposes the interaction of silver nanoparticles with bacteria, either through interacting with bacterial membrane, damaging it and killing the bacteria [27] or by inducing reactive oxygen species (ROS) via interaction with respiratory chain proteins on the membrane and interrupting oxygen reduction. Eventually this ROS causes cellular oxidative stress and bacterial death [28]. Another scenario is based on the adhesion of AgNPs to the bacterial wall, following [27]. It is also reported that small-sized AgNPs cause cytotoxicity in both gram-positive and gram-negative bacteria [29]. Smaller silver nanoparticles have a larger surface area to volume ratio, and they show a faster rate of silver ion dissolution in their microenvironment, thereby inducing greater cytotoxicity than larger NPs [24,30]. The released Ag^+^ ions interact with respiratory chain proteins and induce the production of reactive oxygen species (ROS), thereby causing cellular oxidative stress in microbes and death [24,31] Inside the bacterial cell, silver nanoparticles interact with sulphur and phosphorus, causing DNA replication inhibition. They also inhibit protein synthesis by denaturing ribosomes [7]. Future application of the current results will include the use of nanosilver in coating films used for packaging, in order to keep them sterile and hygienic [32].

## 5. Conclusions

The broad-spectrum antimicrobial effect is due to ease of penetration of AgNPs. The smaller the AgNPs, the more easily they penetrate cells. This clearly shows the potentials of using these biogenic nanoparticles against plant pathogens as an alternative to harmful pesticides.

## Figures and Tables

**Table 1:** Antimicrobial bioassay of three different-sized silver nanoparticles against three plant pathogenic bacteria. Smallest silver nanoparticle average size is 33.9 nm, Medium average size is 67 nm and largest sized is 92.5 nm. Negative control (water) had no inhibition zone whereas positive control inhibition zone diameter was 2.5 cm.

Bacteria	Inhibition Zone of Smallest AgNPs (cm)	Inhibition Zone of Medium AgNPs (cm)	Inhibition Zone of Largest AgNPs (cm)
*Erwinia pyrifoliae*	1.0 ± 0.2	0.8 ± 0.1	0.6 ± 0.1
*Staphylococcus warneri*	1.7 ± 0.1	1.5 ± 0.3	1.0 ± 0.1
*Xanthomonas citri*	1.0 ± 0.1	0.9 ± 0.3	0.9 ± 0.1

**Table 2:** *Erwinia amylovora*.

Incubation Time (Hours)	MIC (% *w*/*v*)				
	0	0.5	1	2	4
0	_a_ 8 ^a^ ± 0.9	_a_ 8 ^a^ ± 0.87	_a_ 8 ^a^ ± 0.77	_a_ 8.1 ^a^ ± 0.91	_a_ 8 ^a^ ± 0.78
0.5	_b_ 7.5 ^a^ ± 0.6	_b_ 7.1 ^b^ ± 0.68	_b_ 6.3 ^c^ ± 0.35	_b_ 5.2 ^d^ ± 0.35	_b_ 3.1 ^e^ ± 0.32
1	_b_ 7.5 ^a^ ± 0.4	_b_ 7 ^b^ ± 0.56	_c_ 5.7 ^c^ ± 0.39	_c_ 4.6 ^d^ ± 0.69	_c_ 2.5 ^e^ ± 0.21
2	_b_ 7.2 ^a^ ± 0.4	_b_ 7 ^b^ ± 0.43	_c_ 5.7 ^c^ ± 0.21	_d_ 3.3 ^d^ ± 0.28	_d_ 0 ^e^ ± 0.0
4	_d_ 7 ^a^ ± 0.22	_c_ 6 ^b^ ± 0.2	_d_ 4.7 ^c^ ± 0.11	_e_ 2.8 ^d^ ± 0.12	_d_ 0 ^e^ ± 0.0
6	_d_ 7 ^a^ ± 0.35	_b_ 6 ^c^ ± 0.4	_e_ 4 ^c^ ± 0.22	_f_ 1.7 ^d^ ± 0.13	_d_ 0 ^e^ ± 0.0

**Table 3:** *Staphylococcus warneri*.

Incubation Time (Hours)	MIC (% *w*/*v*)				
	0	0.5	1	2	4
0	_a_ 8 ^a^ ± 0.8	_a_ 8 ^a^ ± 0.91	_a_ 8 ^a^ ± 0.7	_a_ 8.1 ^a^ ± 0.81	_a_ 8 ^a^ ± 0.8
0.5	_b_ 7.4 ^a^ ± 0.5	_b_ 7 ^b^ ± 0.68	_b_ 6 ^c^ ± 0.5	_b_ 5 ^d^ ± 0.3	_b_ 3 ^e^ ± 0.1
1	_b_ 7.4 ^a^ ± 0.3	_b_ 6.7 ^b^ ± 0.56	_c_ 5.5 ^c^ ± 0.29	_c_ 4.4 ^d^ ± 0.9	_c_ 2.1 ^e^ ± 0.14
2	_c_ 7 ^a^ ± 0.2	_b_ 6.7 ^b^ ± 0.43	_c_ 5.5 ^c^ ± 0.21	_d_ 3.1 ^d^ ± 0.2	_d_ 0 ^e^ ± 0.0
4	_d_ 6 ^a^ ± 0.12	_c_ 6 ^b^ ± 0.2	_d_ 4.2 ^c^ ± 0.1	_e_ 2.2 ^d^ ± 0.1	_d_ 0 ^e^ ± 0.0
6	_d_ 6 ^a^ ± 0.44	_b_ 6 ^c^ ± 0.4	_e_ 4 ^c^ ± 0.12	_f_ 1.5 ^d^ ± 0.1	_d_ 0 ^e^ ± 0.0

**Table 4:** *Xanthomonas citri*.

Incubation Time (Hours)	MIC (% *w*/*v*)				
	0	0.5	1	2	4
0	_a_ 8 ^a^ ± 0.9	_a_ 8 ^a^ ± 0.8	_a_ 8 ^a^ ± 0.1	_a_ 8.1 ^a^ ± 0.1	_a_ 8 ^a^ ± 0.71
0.5	_b_ 7.1 ^a^ ± 0.4	_b_ 7 ^b^ ± 0.2	_b_ 6.1 ^c^ ± 0.4	_b_ 5 ^d^ ± 0.5	_b_ 4.1 ^e^ ± 0.4
1	_b_ 7.1 ^a^ ± 0.3	_b_ 6.6 ^b^ ± 0.4	_c_ 5.1 ^c^ ± 0.29	_c_ 4.5 ^d^ ± 0.9	_c_ 3.5 ^e^ ± 0.3
2	_c_ 7 ^a^ ± 0.3	_b_ 6.6 ^b^ ± 0.3	_c_ 5.1 ^c^ ± 0.22	_d_ 3.2 ^d^ ± 0.8	_d_ 0 ^e^ ± 0.0
4	_d_ 6.5 ^a^ ± 0.1	_c_ 5.5 ^b^ ± 0.1	_d_ 4.3 ^c^ ± 0.1	_e_ 2.4 ^d^ ± 0.3	_d_ 0 ^e^ ± 0.0
6	_d_ 6.5 ^a^ ± 0.5	_b_ 5.5 ^c^ ± 0.3	_e_ 4 ^c^ ± 0.3	_f_ 1.1 ^d^ ± 0.2	_d_ 0 ^e^ ± 0.0

## Data Availability

Not applicable.

## Supplementary Materials

The following supporting information can be downloaded at: https://www.mdpi.com/article/10.3390/antibiotics12071114/s1, Figure S1: UV spectrum of the small sized nanosilver; Figure S2: Uv spectrum of the medium-sized nanosilver; Figure S3: FTIR spectrum of smallest sized (34 nm) biogenic silver nanoparticles; Figure S4: FTIR spectrum of medium-sized (67 nm) biogenic silver nanoparticles; Figure S5: FTIR spectrum of Largest sized (92 nm) biogenic silver nanoparticles; Figure S6: Scanning electron micrograph showing (A) small (average 34 nm), (B) medium (average 67 nm) and (C) large sized (average 92 nm) nanoparticle; Figure S7: The time-kill curve plots of Staphylococcus warneri, Erwinia amylovora and Xanthomonas citri after the exposure to the PT-AgNPs at 0 × MIC, 0.5 × MIC, 1 × MIC, 2 × MIC, and 4 × MIC.

## Abbreviations

AgNPs (Silver nanoparticles); MIC (minimum inhibitory concentration), DMSO (dimethylsulfoxide).

## References

[1] Abou El-Nour K.M., Eftaiha A.A., Al-Warthan A., Ammar R.A. (2010). Synthesis and applications of silver nanoparticles. Arab. J. Chem..

[2] Younis N.S., El Semary N.A., Mohamed M.E. (2021). Silver nanoparticles green synthesis via cyanobacterium *Phormidium* sp.: Characterization, wound healing, antioxidant, antibacterial, and anti-Inflammatory activities. Eur. Rev. Med. Pharam. Sci..

[3] Mohamed M.E., El Semary N.A., Younis N.S. (2022). Silver nanoparticle production by the cyanobacterium *Cyanothece* sp.: De Novo manipulation of nano-biosynthesis by phytohormones. Life.

[4] Lengke M.F., Fleet M.E., Southam G. (2007). Biosynthesis of silver nanoparticles by filamentous cyanobacteria from a silver(I) nitrate complex. Langmuir.

[5] Patel V., Berthold D., Puranik P., Gantar M. (2015). Screening of cyanobacteria and microalgae for their ability to synthesize silver nanoparticles with antibacterial activity. Biotechnol. Rep..

[6] Parial D., Patra H.K., Roychoudhury P., Dasgupta A.K., Pal R. (2012). Gold nanorod production by cyanobacteria—A green chemistry approach. J. Appl. Phycol..

[7] Hamida R.S., Ali M.A., Redhwan A., Bin-Meferij M.M. (2020). Cyanobacteria—A promising platform in green nanotechnology: A Review on nanoparticles fabrication and their prospective applications. Int. J. Nanomed..

[8] Hong X., Wen W.J., Xiong X., Hu Y. (2016). Silver nanowire-carbon fiber cloth nanocomposites synthesized by UV curing adhesive for electrochemical point-of-use water disinfection. Chemosphere.

[9] Pal S., Yu K.T., Song J.M. (2007). Does the antibacterial activity of silver nanoparticles depend on the shape of the nanoparticle? A study of the gram-negative bacterium Escherichia coli. Appl. Environ. Microbiol..

[10] Jeong Y., Lim D., Choi J. (2014). Assessment of Size-Dependent Antimicrobial and Cytotoxic Properties of Silver Nanoparticles. Adv. Mater. Sci. Eng..

[11] Oves M., Rauf M.A., Qari H.A. (2023). Therapeutic Applications of Biogenic Silver Nanomaterial Synthesized from the Paper Flower of *Bougainvillea glabra* (Miami, Pink). Nanomaterials.

[12] More P.R., Pandit S., Filippis A., Franci G., Mijakovic I., Galdiero M. (2023). Silver Nanoparticles: Bactericidal and Mechanistic Approach against Drug Resistant Pathogens. Microorganisms.

[13] Sampath G., Chen Y.-Y., Rameshkumar N., Krishnan M., Nagarajan K., Shyu D.J.H. (2022). Biologically Synthesized Silver Nanoparticles and Their Diverse Applications. Nanomaterials.

[14] Trzcińska-Wencel J., Wypij M., Rai M., Golińska P. (2023). Biogenic nanosilver bearing antimicrobial and antibiofilm activities and its potential for application in agriculture and industry. Front. Microbiol..

[15] Hamida R.S., Abdelmeguid N.E., Ali M.A., Bin-Meferij M.M., Khalil M.I. (2020). Synthesis of Silver Nanoparticles Using a Novel Cyanobacteria *Desertifilum* sp. extract: Their Antibacterial and Cytotoxicity Effects. Int. J. Nanomed..

[16] Rippka R., Deruelles J., Waterbury J.B., Herdman M., Stanier R.Y. (1979). Generic assignments, strain histories and properties of pure cultures of cyanobacteria. J. Gen. Microbiol..

[17] El Semary N., Bakir E. (2022). Multidrug-resistant bacterial pathogens and public health: The antimicrobial effect of cyanobacterial-biosynthesized silver nanoparticles. Antibiotics.

[18] Patra J.K., Baek K.-H. (2017). Antibacterial Activity and Synergistic Antibacterial Potential of Biosynthesized Silver Nanoparticles against Foodborne Pathogenic Bacteria along with its Anticandidal and Antioxidant Effects. Front. Microbiol..

[19] Kubo I., Fujita K., Kubo A., Nihei K., Ogura T. (2004). Antibacterial activity of coriander volatile compounds against Salmonella choleraesuis. J. Agric. Food Chem..

[20] Dong Y., Zhu H., Shen Y., Zhang W., Zhang L. (2019). Antibacterial activity of silver nanoparticles of different particle size against Vibrio natriegens. PLoS ONE.

[21] Zhang X.-F., Liu Z.-G., Shen W., Gurunathan S. (2016). Silver Nanoparticles: Synthesis, Characterization, Properties, Applications, and Therapeutic Approaches. Int. J. Mol. Sci..

[22] Mota R., Flores C., Tamagnini P., Oliveira J.M., Radhouani H., Reis R.L. (2021). Cyanobacterial Extracellular Polymeric Substances (EPS). Polysaccharides of Microbial Origin.

[23] Gharibshahi L., Saion E., Gharibshahi E., Shaari A.H., Matori K.A. (2017). Structural and Optical Properties of Ag Nanoparticles Synthesized by Thermal Treatment Method. Materials.

[24] Ferdous Z., Nemmar A. (2020). Health impact of silver nanoparticles: A Review of the biodistribution and toxicity following various routes of exposure. Int. J. Mol. Sci..

[25] Durán N., Silveira C.P., Durán M., Martinez D.S.T. (2015). Silver nanoparticle protein corona and toxicity: A mini-review. J. Nanobiotechnol..

[26] Ali D.M., Sasikala M., Gunasekaran M., Thajuddin N. (2011). Biosynthesis and characterization of silver nanoparticles using marine cyanobacterium, Oscillatoria willei NTDM01. Dig. J. Nanomater. Biostruct..

[27] Qing Y., Cheng L., Li R., Liu G., Zhang Y., Tang X., Wang J., Liu H., Qin Y. (2018). Potential antibacterial mechanism of silver nanoparticles and the optimization of orthopedic implants by advanced modification technologies. Int. J. Nanomed..

[28] Lee S.H., Jun B.-H. (2019). Silver nanoparticles: Synthesis and application for nanomedicine. Int. J. Mol. Sci..

[29] Das R., Gang S., Nath S.S. (2011). Preparation and antibacterial activity of silver nanoparticles. J. Biomater. Nanobiotechnol..

[30] Shang L., Nienhaus K., Nienhaus G.U. (2014). Engineered nanoparticles interacting with cells: Size matters. J. Nanobiotechnol..

[31] Long Y.-M., Hu L.-G., Yan X.-T., Zhao X.-C., Zhou Q.-F., Cai Y., Jiang G.-B. (2017). Surface ligand controls silver ion release of nanosilver and its antibacterial activity against Escherichia coli. Int. J. Nanomed..

[32] Baysal G., Demirci C., Özpinar H. (2023). Proporties and synthesis of biosilver nanofilms for antimicrobial food packaging. Polymers.

