# Supplementary material for: Differential Antimicrobial Effect of Three-Sized Biogenic Silver Nanoparticles as Broad-Spectrum Antibacterial Agents against Plant Pathogens
Source: Antibiotics (Basel). 2023 Jun 28;12(7):1114. doi: 10.3390/antibiotics12071114 (PMC10376758; doi:10.3390/antibiotics12071114)
Supplement: Supplementary file 1 [file antibiotics-12-01114-s001.zip › antibiotics-2335568-supplementary.pdf]

Supplementary materials

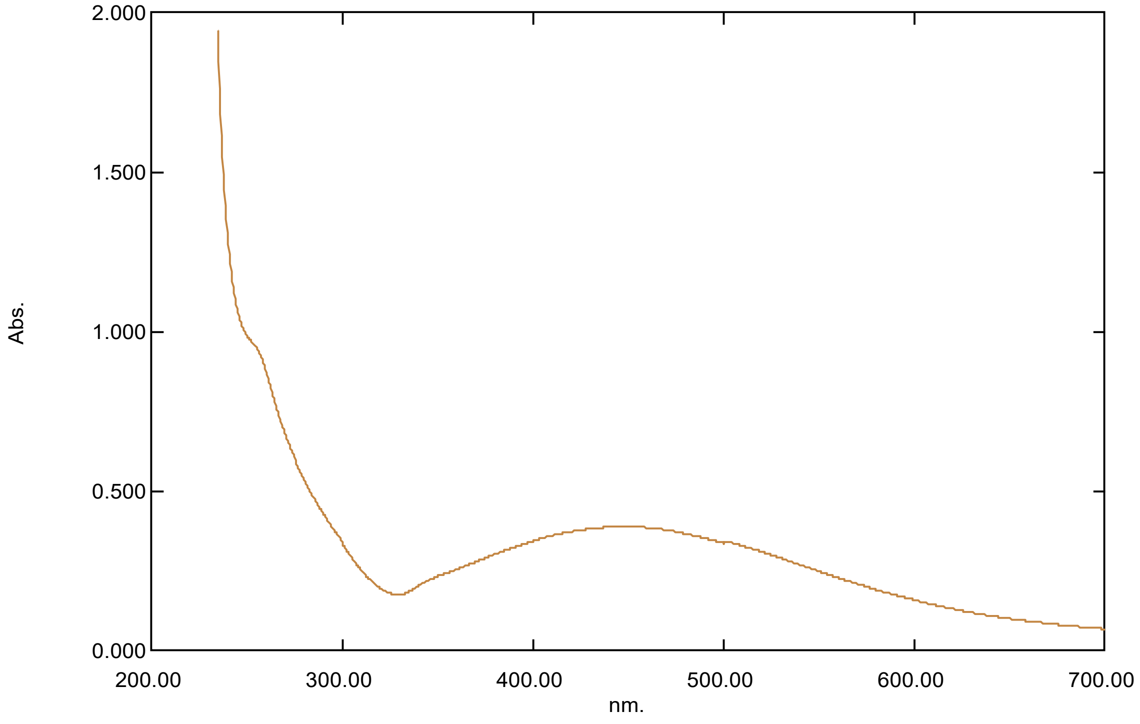

Figure S1. UV spectrum of the small sized nanosilver

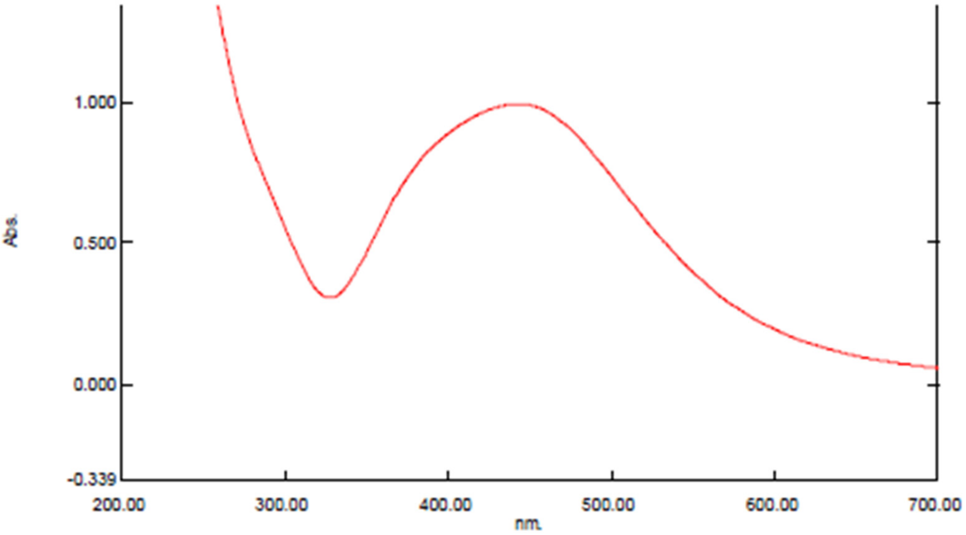

Figure S2. Uv spectrum of the medium-sized nanosilver

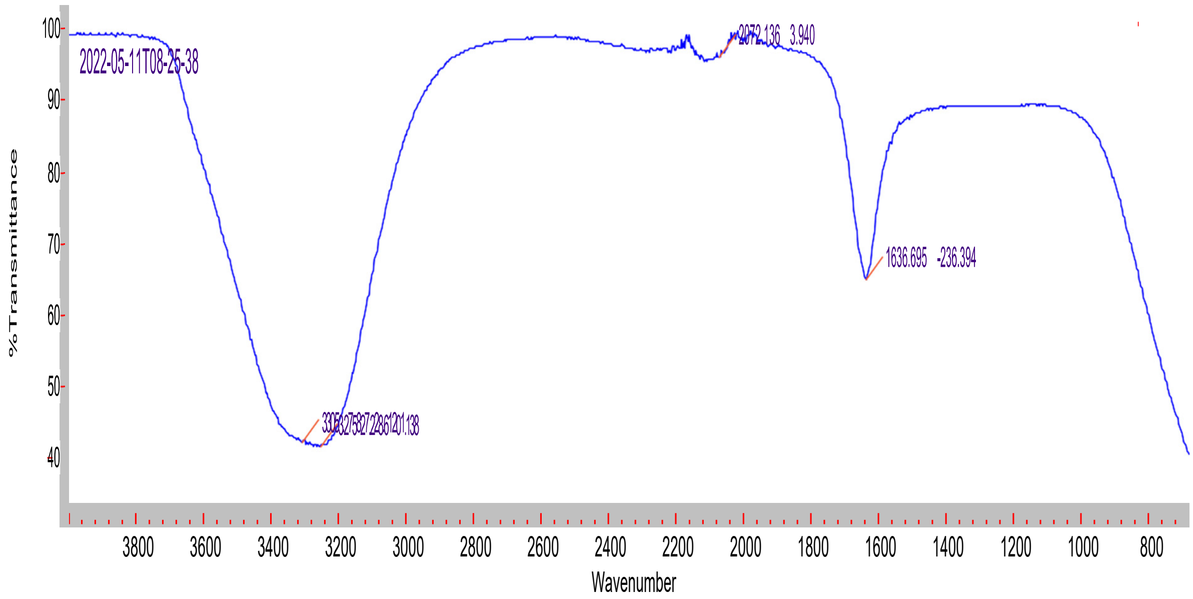

Figure S3. FTIR spectrum of smallest sized (34nm) biogenic silver nanoparticles. X-axis denotes wave number whereas Y axis denotes % transmittance

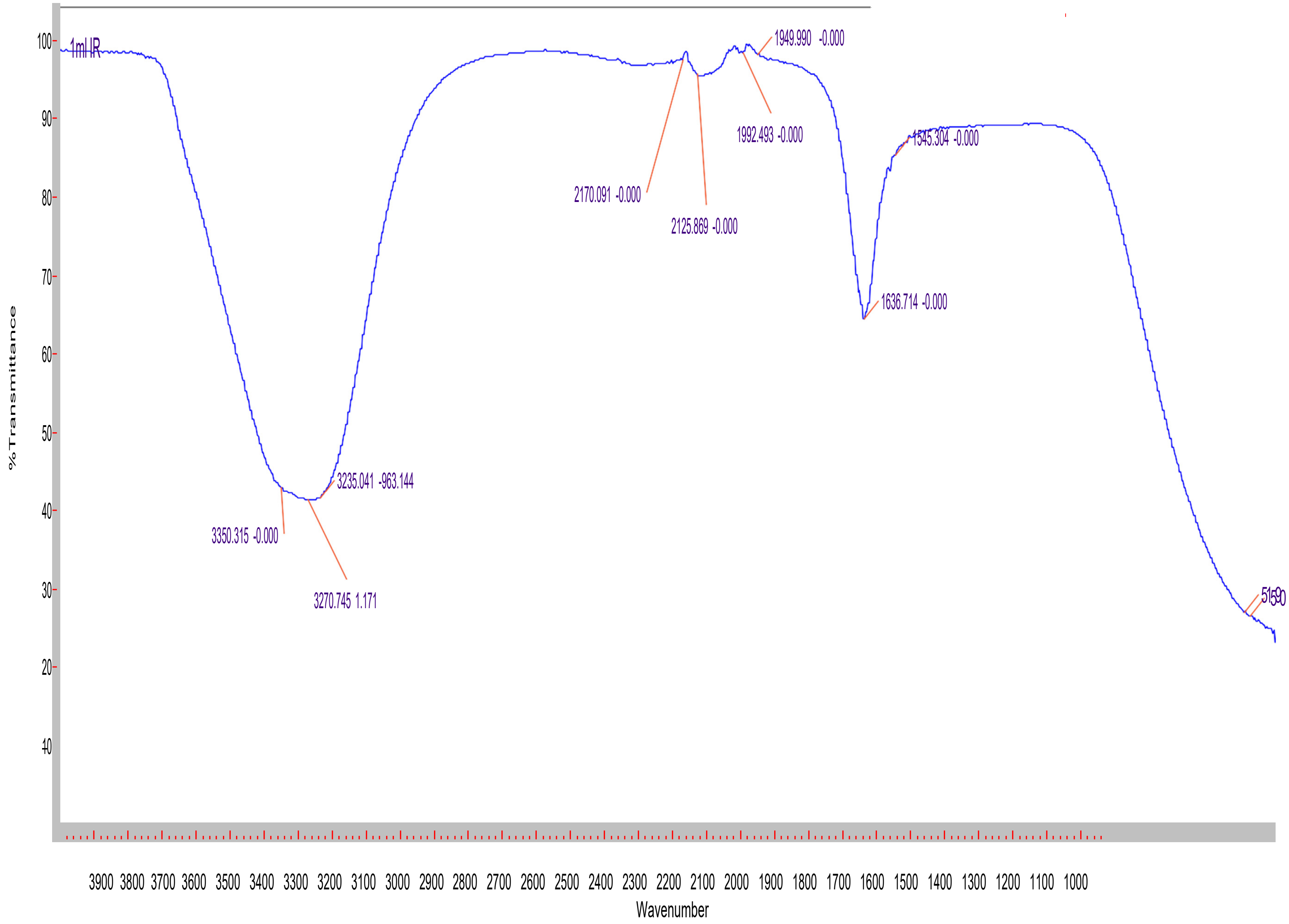

Figure S4. FTIR spectrum of medium- sized (67 nm) biogenic silver nanoparticles. X-axis denotes wave number whereas Y axis denotes % transmittance

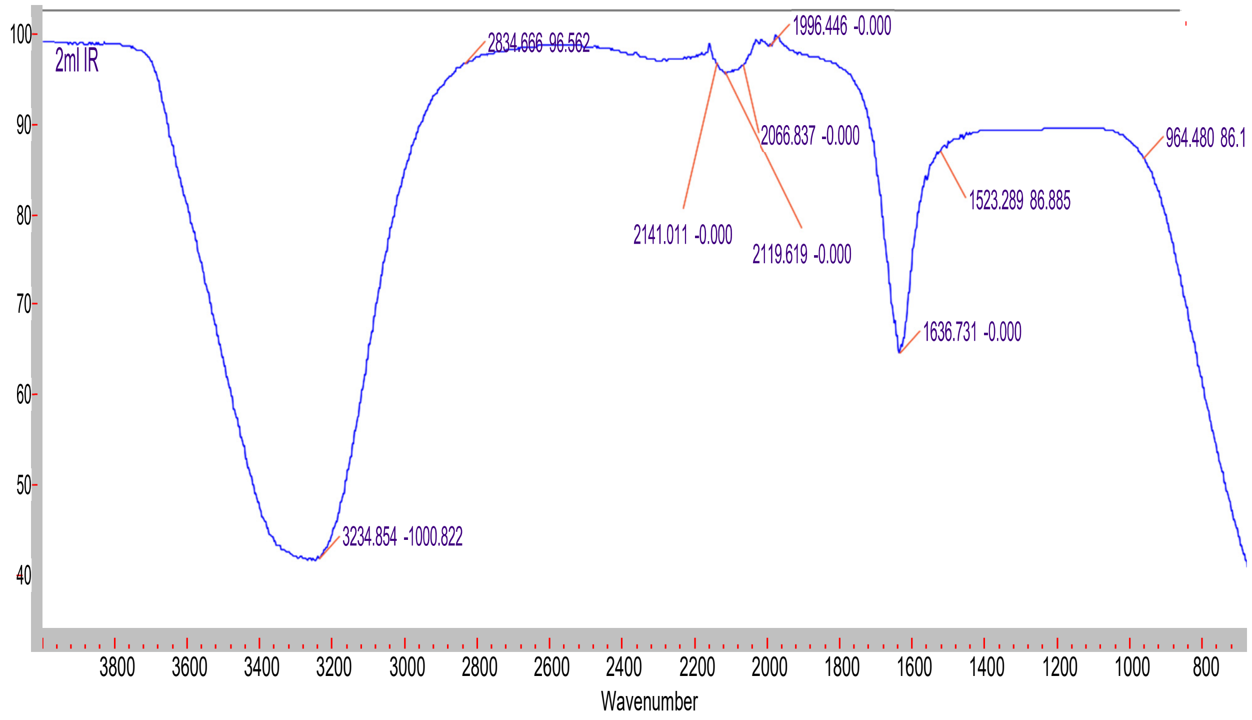

Figure S5. FTIR spectrum of Largest sized (92nm) biogenic silver nanoparticles. X-axis denotes wave number whereas Y axis denotes % transmittance.

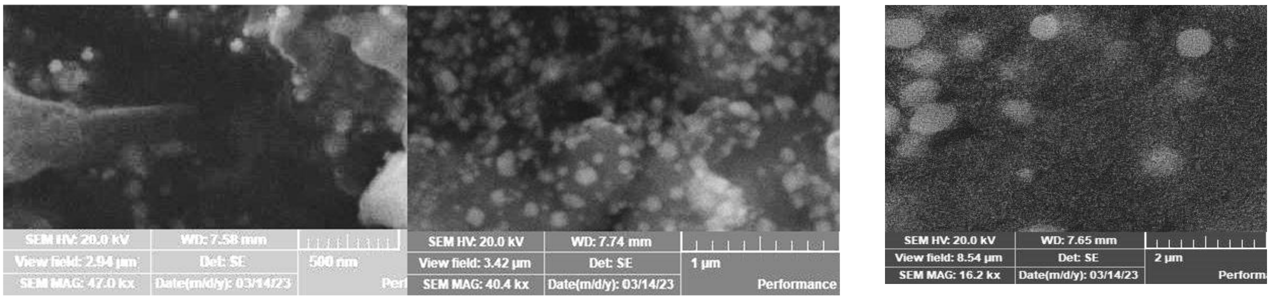

(**A**) (**B**) (**C**)

Figure S6. Scanning electron micrograph showing (A) small (average 34nm), (B) medium (average 67 nm) and (C) large sized (average 92 nm) nanoparticle. Scale bar (20 nm).

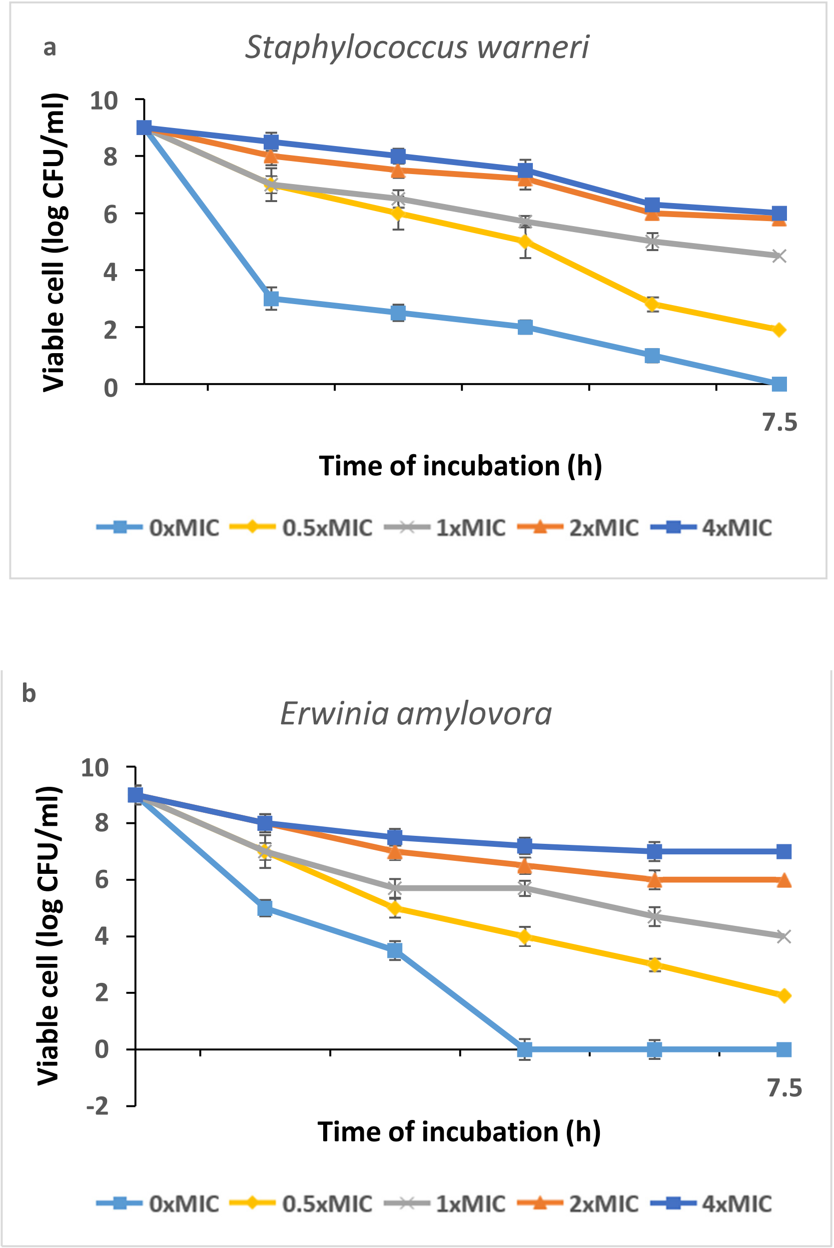
a
Staphylococcus warneri
Viable cell (log CFU/ml)
10
8
6
4
2
0
7.5
Time of incubation (h)
0xMIC
0.5xMIC
1xMIC
2xMIC
4xMIC
b
Erwinia amylovora
Viable cell (log CFU/ml)
10
8
6
4
2
0
-2
7.5
Time of incubation (h)
0xMIC
0.5xMIC
1xMIC
2xMIC
4xMIC

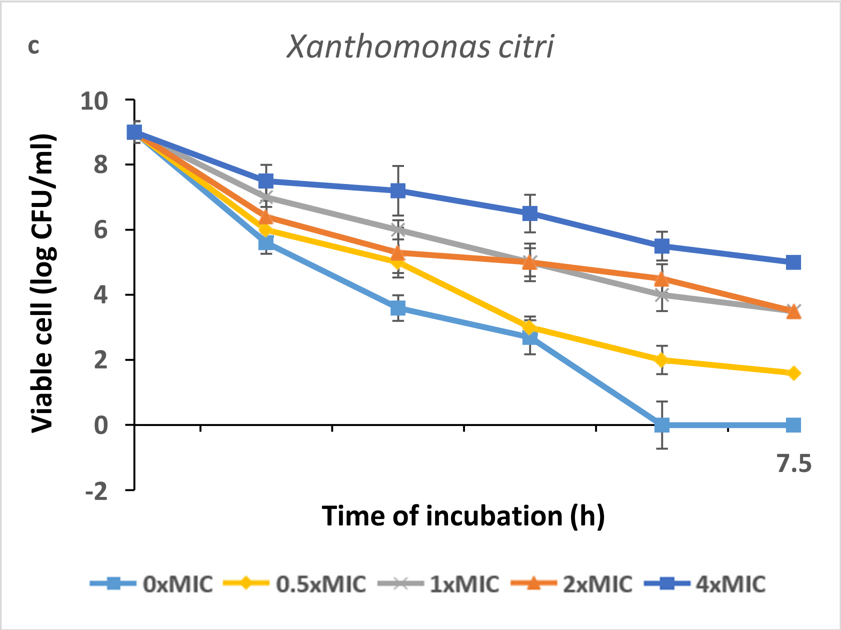

Figure S7 (a, b and c). The time-kill curve plots of *Staphylococcus warneri*, *Erwinia amylovora* and *Xanthomonas citri* after the exposure to the PT-AgNPs at 0 × MIC, 0.5 × MIC, 1 × MIC, 2 × MIC, and 4× MIC. The test was performed using the largest sized nanosilver (92 nm)
